# Phenotypic Characterization of Bone Marrow Mononuclear Cells and Derived Stromal Cell Populations from Human Iliac Crest, Vertebral Body and Femoral Head

**DOI:** 10.3390/ijms20143454

**Published:** 2019-07-14

**Authors:** Marietta Herrmann, Maria Hildebrand, Ursula Menzel, Niamh Fahy, Mauro Alini, Siegmund Lang, Lorin Benneker, Sophie Verrier, Martin J. Stoddart, Jennifer J. Bara

**Affiliations:** 1 AO Research Institute Davos, Clavadelerstrasse 8, 7270 Davos Platz, Switzerland; 2 IZKF Group Tissue Regeneration in Musculoskeletal Diseases, University Clinics Wuerzburg, 97070 Wuerzburg, Germany; 3 Orthopedic Center for Musculoskeletal Research, University of Wuerzburg, 97076 Wuerzburg, Germany; 4 Department of Orthopaedics, Erasmus MC, University Medical Center Rotterdam, 3015 Rotterdam, The Netherlands; 5 Department of Oral and Maxillofacial Surgery, Erasmus MC, University Medical Center Rotterdam, 3015 Rotterdam, The Netherlands; 6 Department of Trauma Surgery, Regensburg University Medical Center, 93053 Regensburg, Germany; 7 Department of Orthopedic Surgery and Traumatology, Spine Unit, Inselspital, Bern University Hospital, 3010 Bern, Switzerland; 8 Department of Orthopaedic Surgery, Washington University, St Louis, MO 63110, USA

**Keywords:** bone marrow stromal cells, MSC, pericytes, femoral head, vertebral body, iliac crest, chondrogenesis

## Abstract

(1) In vitro, bone marrow-derived stromal cells (BMSCs) demonstrate inter-donor phenotypic variability, which presents challenges for the development of regenerative therapies. Here, we investigated whether the frequency of putative BMSC sub-populations within the freshly isolated mononuclear cell fraction of bone marrow is phenotypically predictive for the in vitro derived stromal cell culture. (2) Vertebral body, iliac crest, and femoral head bone marrow were acquired from 33 patients (10 female and 23 male, age range 14–91). BMSC sub-populations were identified within freshly isolated mononuclear cell fractions based on cell-surface marker profiles. Stromal cells were expanded in monolayer on tissue culture plastic. Phenotypic assessment of in vitro derived cell cultures was performed by examining growth kinetics, chondrogenic, osteogenic, and adipogenic differentiation. (3) Gender, donor age, and anatomical site were neither predictive for the total yield nor the population doubling time of in vitro derived BMSC cultures. The abundance of freshly isolated progenitor sub-populations (CD45−CD34−CD73+, CD45−CD34−CD146+, NG2+CD146+) was not phenotypically predictive of derived stromal cell cultures in terms of growth kinetics nor plasticity. BMSCs derived from iliac crest and vertebral body bone marrow were more responsive to chondrogenic induction, forming superior cartilaginous tissue in vitro, compared to those isolated from femoral head. (4) The identification of discrete progenitor populations in bone marrow by current cell-surface marker profiling is not predictive for subsequently derived in vitro BMSC cultures. Overall, the iliac crest and the vertebral body offer a more reliable tissue source of stromal progenitor cells for cartilage repair strategies compared to femoral head.

## 1. Introduction

Adult bone marrow contains a heterogeneous population of stromal cells, bone marrow-derived stromal cells (BMSCs) (also referred to as mesenchymal stromal cells (MSCs) or skeletal progenitor cells), which may be isolated in vitro and differentiated toward chondrogenic, osteogenic, and adipogenic cell lineages [1,2,3,4]. Recent lineage tracing and fate mapping studies suggest that, apart from being phenotypically heterogeneous, these bone marrow stromal cells are also developmentally distinct. Evidence points not only to the somatic lateral plate mesoderm [5,6] but also to neural crest origins [7]. 

In vitro selected and culture expanded BMSCs have historically been characterized by their adherence to tissue culture plastic, multipotent differentiation potential, and cluster of differentiation (CD) marker profile: ≥95% CD90+, CD73+, CD105+ and ≥95% CD45−, CD34−, CD14/CD11b, CD79α/CD19−, HLA−DR− [8]. Despite this commonly shared set of criteria, genotypic and phenotypic variation between BMSC cultures is very prominent. Inter- and intra-donor variations are widely reported and present challenges not only for biological investigations but also as we strive to develop efficacious cell-based therapies. 

Studies characterizing the colony forming unit (CFU) fraction from freshly isolated mononuclear cells (MNCs) reveal that the classical BMSC cell surface marker profile is in fact largely acquired during monolayer culture [9,10,11,12]. It is now clear that freshly isolated BMSC populations exhibit a more diverse cell surface marker expression profile than previously realized. Indeed, BMSCs are an inherently heterogeneous population of cells occupying spatially and functionally discrete endosteal, stromal, and perivascular niches [13,14,15]. Rasini et al., 2013 reported site-specific phenotypes based on cell-surface marker expression between cells in different anatomical localizations [16]. Previous studies focused on characterizing individual BMSC populations in human bone marrow within the freshly isolated MNC fraction: CD45^low^/D7FIB+/CD271+ [11]; lin−/CD271+/CD45−/CD146+ [17]; CD64^bright^/CD31^bright^/CD14^neg^ [18]; CD13^high^/CD105+/CD45− [19]; CD145−/CD34−/CD146+ [20,21,22]; PDPN+/CD146−CD73+CD164+ [23]. Whether the abundance of these discrete BMSC subsets may determine the yield and/or the phenotypic characteristics of the subsequently in vitro derived stromal population remains unclear.

Several groups have investigated the influence of donor demographics, particularly donor age, on the phenotypic variation observed in BMSC cultures with inconsistent results. Whilst some studies suggest a decrease in CFU formation efficiency with age [24,25], others suggest that progenitor cell numbers are maintained [26,27]. With regards to proliferation capacity, it was reported that there is a significant decline in maximal population doublings achieved in cultures from older donors [27,28], and accordingly that population doubling time increases [26] with age. Conversely, others report no age-related differences in maximal population doublings [29] or population doubling time [30]. Contradictory results in terms of plasticity are apparent, with some groups reporting that the ability of BMSCs to differentiate osteogenically, adipogenically, and chondrogenically in vitro is not influenced by donor age [26,30,31]. In contrast, an age-related decline in MSC chondrogenesis from male donors was reported [32]. 

BMSCs may be isolated from several bone marrow sources—vertebral body [33], iliac crest [34], and the femoral head [35]. Although BMSC phenotype has been described in the case of these respective tissue sources, differences in isolation and culture protocols between research groups (which is known to cause phenotypic shift) prevents reliable comparison [36]. Previous groups have compared stromal cells derived from vertebral body vs. iliac crest [34,37]. As far as we are aware, there are no reports of a single study directly comparing derived BMSC populations from vertebral body, iliac crest, and femoral head. We hypothesized that the abundance of progenitor cell populations from these tissues and the behavior of derived BMSC cultures may be different. The aim of this study was to ascertain whether the abundance of specific BMSC subpopulations present in the initial MNC fraction is predictive of the yield and the phenotype of in vitro derived BMSC cultures. In addition to this, we directly compared cell populations from different bone marrow tissue sources and investigated the effect of donor demographics. MNCs were characterized by applying previously reported cell surface marker panels identifying BMSC populations; CD45−CD34−CD73+ [38], CD45−CD34−CD146+ [20,21,22] and pericytes CD146+NG2+ [39]. The yield, the growth kinetics, and the multipotency of derived BMSC cultures were compared between different tissue sources and correlated to the relative abundance of freshly isolated progenitor cells.

## 2. Results

### 2.1. BMSC Yield and Growth Kinetics

**Figure 1:**
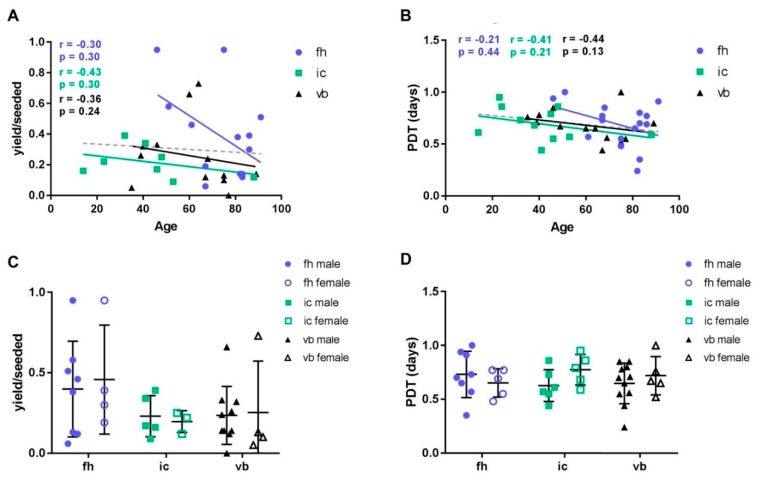
Growth kinetics of BMSCs from different bone marrow sources. (**A**,**C**) The number of cells obtained at p0 in relation to originally seeded MNCs (yield/seeded) was recorded as a measure of initial frequency of MSCs. (**B**,**D**) The population doubling time (PDT) between p0 and p1 is shown as the measure of the proliferation capacity of MSCs. (**A**,**B**) Donor age (correlation tested with Spearman rank test, individually calculated for each tissue source and for the complete data set (linear regression depicted as dashed line; yield: *r* = −0.17, *p* < 0.34; PDT: *r* = −0.23, *p* = 0.15)), (**C**,**D**) bone marrow source (fh: femoral head, ic: iliac crest, vb: vertebral body) and gender did not affect MSC yield or PDT. Kruskal–Wallis statistics with Dunn’s multiple comparison test was performed to test for differences in yield/seeded and PDT, respectively, between different tissue sources. *n* = 33.

In total, we analyzed BMSCs from 33 donors, whereof 15 cultures were isolated from femoral head-derived bone marrow, eight from iliac crest, and 10 from vertebral body (Table 1). All BMSCs exhibited a typical fibroblastic morphology when cultured on tissue culture plastic. We assessed cell yield upon first passaging in relation to the number of originally seeded mononuclear cells. There was no correlation between BMSC yield at first passage and donor age (Figure 1A). No significant differences were observed when comparing different anatomical sites or between male and female donors (Figure 1C). Following subsequent culture expansion, variation in growth kinetics between donors was apparent and was characteristic of primary human BMSCs. No correlation was seen between donor age and the number of population doublings/day in BMSC cultures between p0–p1 (Figure 1B). Furthermore, there were no significant differences in the mean number of population doublings/day between BMSC cultures when comparing the three tissue sources of bone marrow or male and female donors (Figure 1D).

### 2.2. Identification of Putative Progenitor Cell Subsets within the Freshly Isolated MNC Fraction

**Figure 2:**
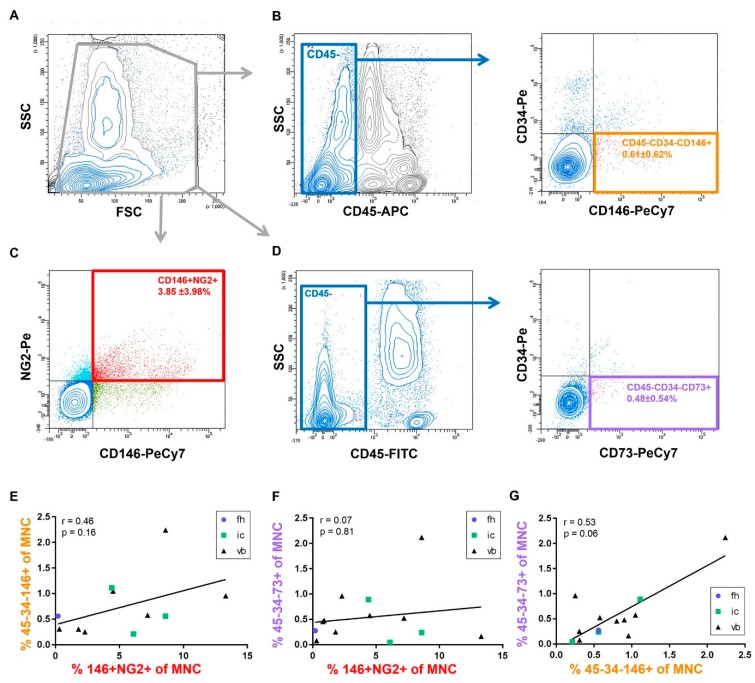
Detection of putative progenitor populations within the freshly isolated mononuclear cell fraction. Cell-surface marker panels were applied to identify putative progenitor populations. (**A**) Mononuclear cells were gated by forward scatter (FSC)–site scatter (SSC) profile. (**B**) Gating strategy for the detection of CD34−CD146+ cells in the CD45− cell fraction (orange). (**C**) Identification of cells co-expressing CD146 and NG-2 (red). (**D**) Gating strategy for the detection of CD34−CD73+ cells in the CD45− cell population (violet). (**E**–**G**) The relative abundance of specific progenitor sub-populations were plotted against each other correlated by Spearman rank, tissue source of samples as indicated (fh = femoral head, ic = iliac crest, vb = vertebral body). *n* = 11 (**E**); *n* = 13 (**F**,**G**).

Identification of freshly isolated BMSCs in the MNC fraction is challenging due to low abundance and the lack of specific cell surface markers. Here, we applied three different marker panels to detect putative BMSC populations (Figure 2). We applied an established marker panel for perivascular progenitor cells, CD45−CD34−CD146+ [20], in addition to a dual stain for pericyte cell surface markers, CD146 and NG2 [39]. Finally, in accordance with published literature and our own, we identified the CD45−CD34−CD73+ MSC subset, which is reported at perivascular, endosteal, and stromal sites [11,12,16,38]. As expected, BMSCs identified by putative cell-surface marker panels were present at very low abundance within the MNC fraction across all tissue sources (Figure 2A–D: CD45−CD34−CD146+: 0.61 ± 0.62%, CD45−CD34−CD73+: 0.48 ± 0.54%, CD146+NG2+: 3.85% ± 3.98). The relative abundances of the above BMSC populations were plotted against each other in order to examine marker panel correspondence (Figure 2E–G). The abundances of CD45-CD34-CD146+ and CD45−CD34−CD73+ populations correlated, although this was not statistically significant (Figure 2G). In contrast, dual staining for CD146 and NG2 detected a larger proportion of MNCs (Figure 2C: 3.85 ± 3.98%), which did not correlate with either CD45−CD34−CD146+/CD45−CD34−CD73+ subsets (Figure 2E,F).

### 2.3. Progenitor Abundance in the Freshly Isolated Mononuclear Cell Fraction Is Not Predictive of the Derived Monolayer Expanded BMSC Population

**Figure 3:**
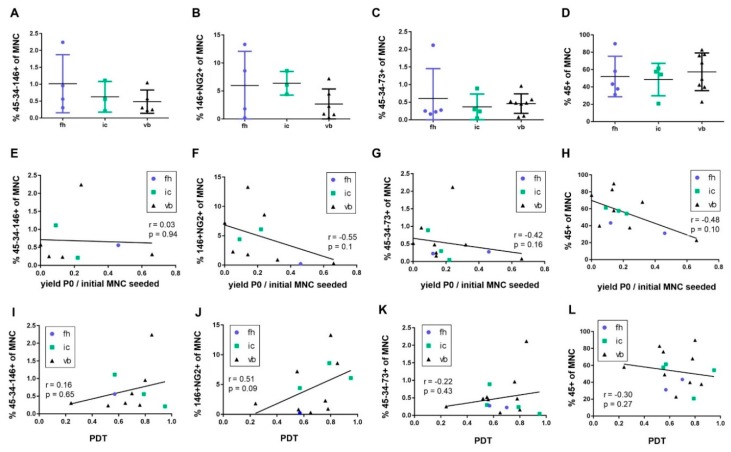
Abundance of putative progenitor populations from different bone marrow sources and correlation analyses. Comparison of the frequency of MSC populations, identified as (**A**) CD45−CD34−CD146+ (*n* = 12), (**B**) CD146+NG2+ (*n* = 13), or (**C**) CD45−CD34−CD73+ (*n* = 17) and (**D**) CD45+ leukocytes (*n* = 17) in bone marrow (BM)-derived MNCs from different sources. Frequency of all cell populations was variable in individual donors but not significantly different in-between tissue sources (fh = femoral head, ic = iliac crest, vb = vertebral body), as assessed by Kruskal–Wallis statistics with Dunn’s multiple comparison test. (**E**–**H**) The relative abundance of BMSC populations was correlated by Spearman rank to the total yield of cells derived as a proportion of the initial number of MNCs originally seeded. (**I**–**L**) The abundance of BMSC populations was further correlated by Spearman rank to the proliferation potential of the cells upon monolayer expansion, as shown by PDT.

The abundance of progenitor populations within the MNC fraction from different bone marrow sources was assessed (Figure 3A–D). Inter-donor variability was apparent with no statistically significant difference in progenitor abundance, as determined by our marker panels, between different anatomical sites. We next investigated whether MNC fractions containing a higher frequency of putative progenitor populations would give rise to higher BMSC yields following monolayer expansion. The abundance of CD45−CD34−CD146+, CD146+/NG2+, or CD45−CD34−CD73+ populations in the freshly isolated MNC fraction did not correlate with the yield of BMSCs at p0 (Figure 3E–G). No significant correlations were apparent between the frequency of progenitors in the freshly isolated MNC fraction and the population doubling time of the subsequently derived stromal population in the analyzed samples, which were mainly derived from vertebral body (Figure 3I–K). We observed a trend between the abundance of CD146+NG2+ cells and the population doubling time; however, this was not statistically significant (*p* = 0.09, Figure 3J). We further analyzed CD45 (marker of the haematopoietic lineage) to test whether the abundance of haematopoietic cells in the initial MNC population might affect the growth kinetics of BMSCs. We found a trend of a negative correlation of the abundance of CD45+ leukocytes in the MNC fraction with the BMSC yield in p0, which was also not statistically significant (*p* = 0.10, Figure 3H). No correlation was observed between the abundance of CD45+ in the MNC fraction and the population doubling time (Figure 3L).

### 2.4. Immunophenotyping of BMSCs

BMSC lines adopted a classical cell-surface marker profile following selection and expansion on tissue culture plastic, as shown by positive expression for CD44, CD90, CD73, and CD105 (Table 2). Whilst CD146 was robustly expressed on cells derived from the iliac crest (ic) and the vertebral body (vb), expression levels were more variable at p0 and p1 in MSCs from the femoral head (fh) (fh: 66.43 ± 22.69%, ic: 97.38 ± 2.39%, vb: 95.07 ± 2.92% CD146+ positive cells in p1, Table 2). NG2 was detected at variable levels in culture expanded MSC populations from across tissue sources, which was also reflected in the variable abundance of CD146+/NG2+ double positive cells.

### 2.5. Tri-Lineage In Vitro Differentiation

**Figure 4:**
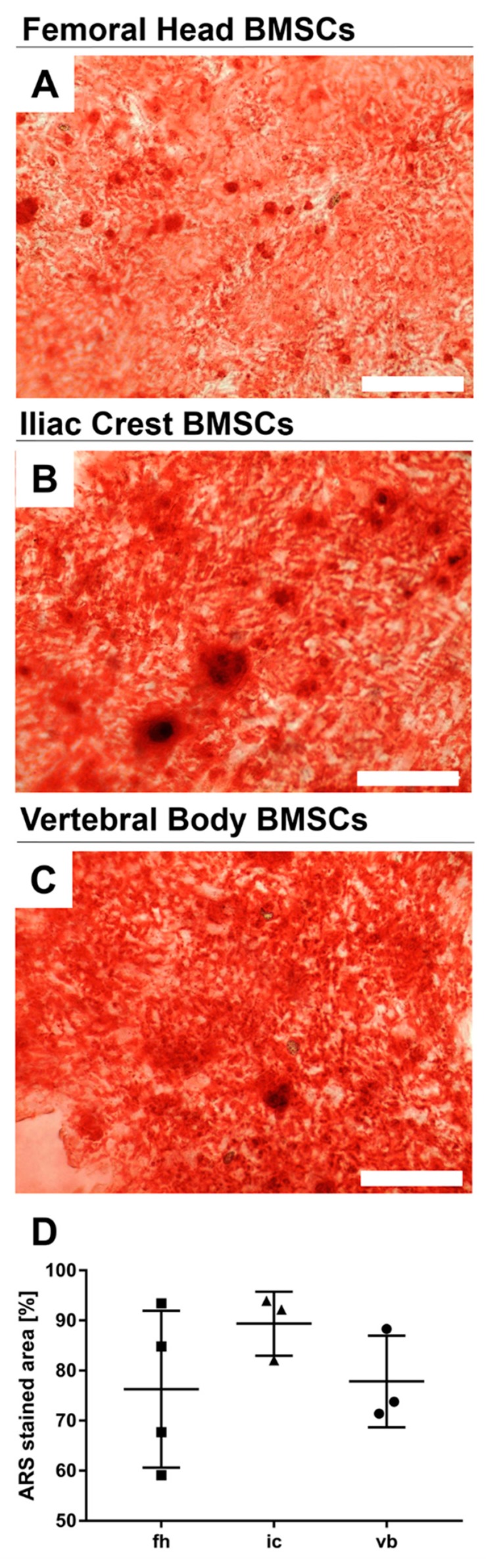
Osteogenic differentiation of BMSCs from different bone marrow sources. Representative images of osteogenically differentiated BMSCs from (**A**) femoral head, (**B**) iliac crest, (**C**) vertebral body assessed by Alizerin Red (ARS) staining. Deposited mineral appears red stained. (**D**) Scans of the entire stained wells were used for quantification of the stained area by densitometry. Data shown are mean values ± standard deviation; no significant differences were detected as assessed by Kruskal–Wallis statistics with Dunn’s multiple comparison test. Scale bars 400 µm. *n* = 10.

In vitro tri-lineage differentiation potential was tested for at least three BMSC donors from each anatomical site. BMSCs from all anatomical sites readily mineralized, as shown by Alizarin Red S (ARS) staining of monolayer cultures after three-week induction with osteogenic medium (Figure 4A–C). Image quantification revealed no significant differences in the area of positive ARS staining between the analyzed donors, although iliac crest derived BMSCs appeared to mineralize more readily and reproducibly compared to femoral head and vertebral body derived cells (Figure 4D).

**Figure 5:**
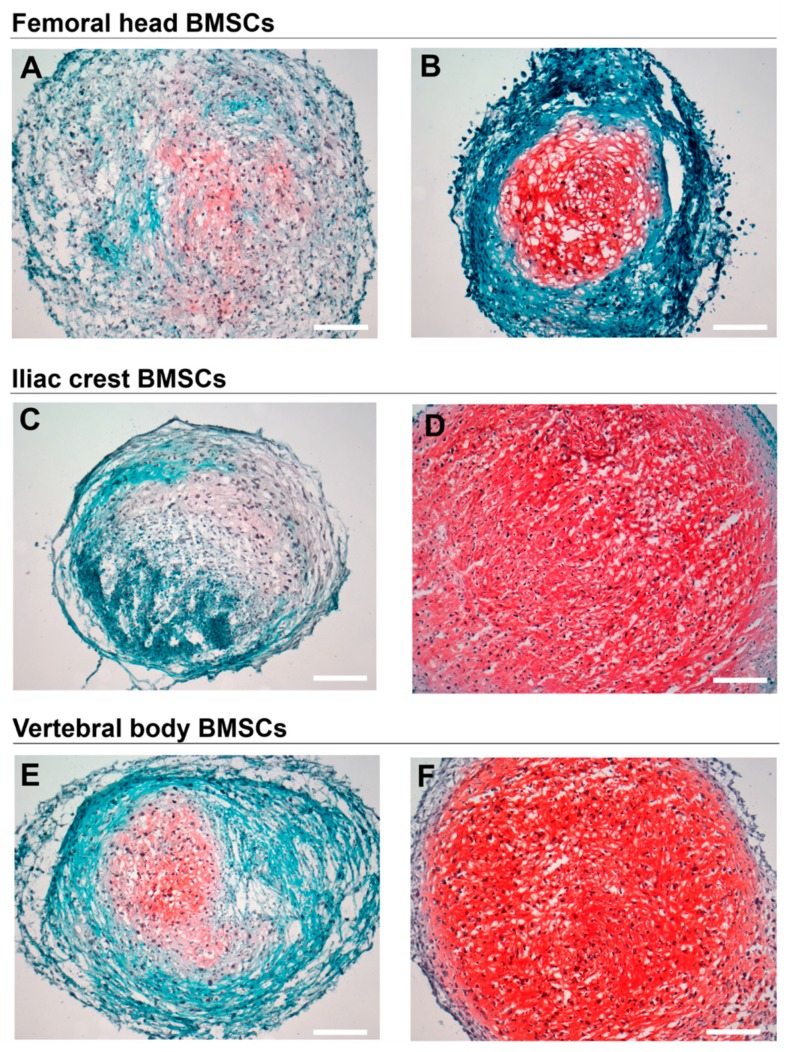
Histological analysis of chondrogenically differentiated BMSCs. Safranin-O Fast Green Haematoxylin staining depicting sulphated-glycosaminoglycan content of chondrogenic pellet cultures. Representative images are presented from the least (**A**,**C**,**E**) and the most (**B**,**D**,**F**) responsive chondrogenic BMSC donors derived from (**A**,**B**) femoral head, (**C**,**D**) iliac crest, and (**E**,**F**) vertebral body bone marrow. Scale bars 200 µm. *n* = 9.

**Figure 6:**
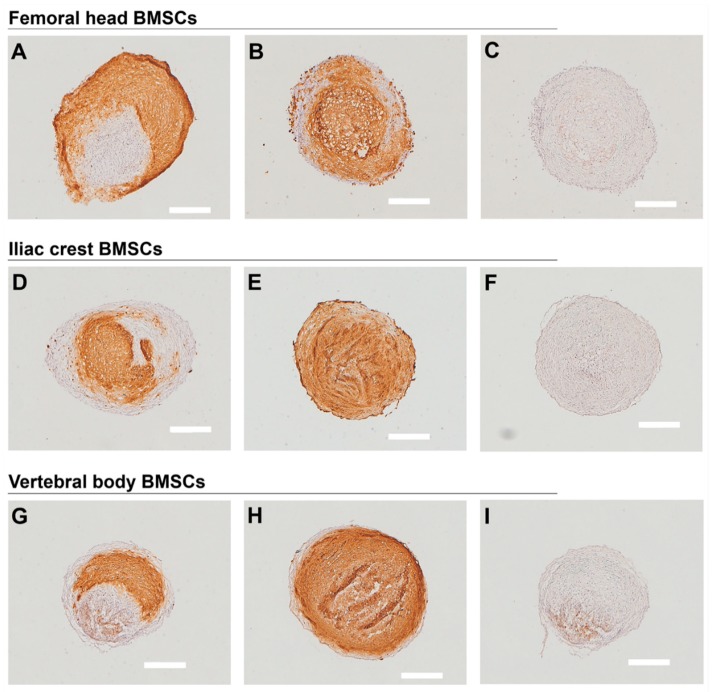
Immunohistochemical detection of collagen type II. Representative images of collagen type II immunostaining are presented from the least (**A**,**D**,**G**) and the most (**B**,**E**,**H**) responsive chondrogenic BMSC donors derived from (**A**–**C)** femoral head, (**D**–**F**) iliac crest, (**G**–**I**) vertebral body bone marrow. (**C**,**F**,**I**) Negative controls. Scale bars 400 µm. *n* = 9.

**Figure 7:**
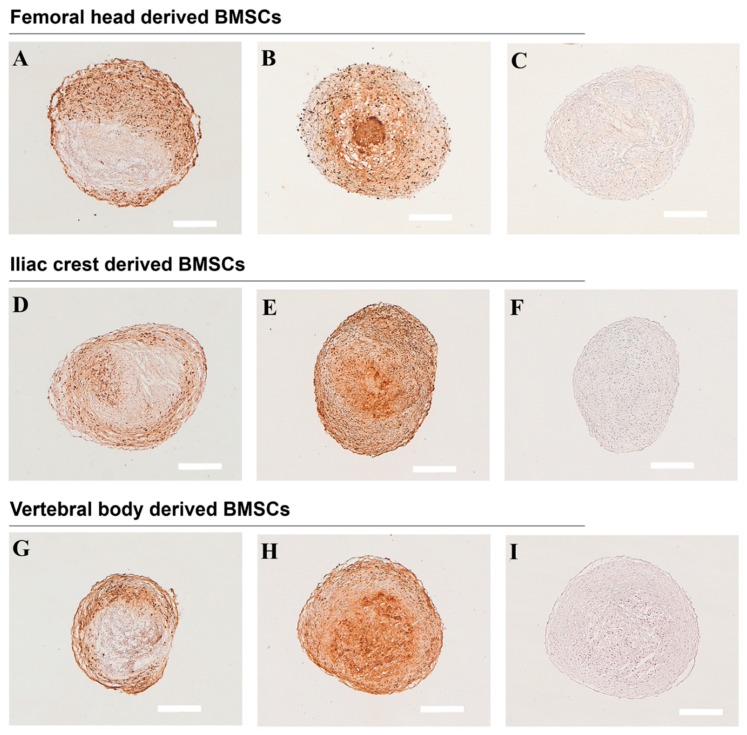
Immunohistochemical detection of collagen type X. Representative images of collagen type X immunostaining are presented from the least (**A**,**D**,**G**) and the most (**B**,**E**,**H**) responsive chondrogenic BMSC donors derived from (**A**–**C**) femoral head, (**D**–**F**) iliac crest, (**G**–**I**) vertebral body bone marrow. (**C**,**F**,**I**) Negative controls. Scale bars 400 µm. *n* = 9.

All BMSC sources formed physically stable chondrogenic pellets. Pellet size varied between donors of all three bone marrow tissue sources (Table 3). Overall, BMSC donors analyzed here derived from iliac crest (Figure 5B,C) and vertebral body (Figure 5E,F) formed larger pellets compared to femoral head (Figure 5A). Representative images showing the full range of histological and immunohistochemical staining (i.e., the least and the most responsive chondrogenic pellets) are shown in Figure 5, Figure 6, and Figure 7. The sulphated glycosaminoglycan (sGAG) content of the extracellular matrix varied between donors, as shown by Safranin-O staining, indicating a pattern of responders and non-responders to TGFbeta-1 induced chondrogenesis (Figure 5, Table 3). In donors responsive to chondrogenic induction, sGAG content appeared higher overall in cultures derived from iliac crest and vertebral body compared to femoral head. Both type II (Figure 6) and type X collagen (Figure 7) were detected immunohistochemically in all donors. In accordance with sGAG content, responding chondrogenic donors exhibited greater immunostaining for collagens II and X compared to the poor chondrogenic cells.

**Figure 8:**
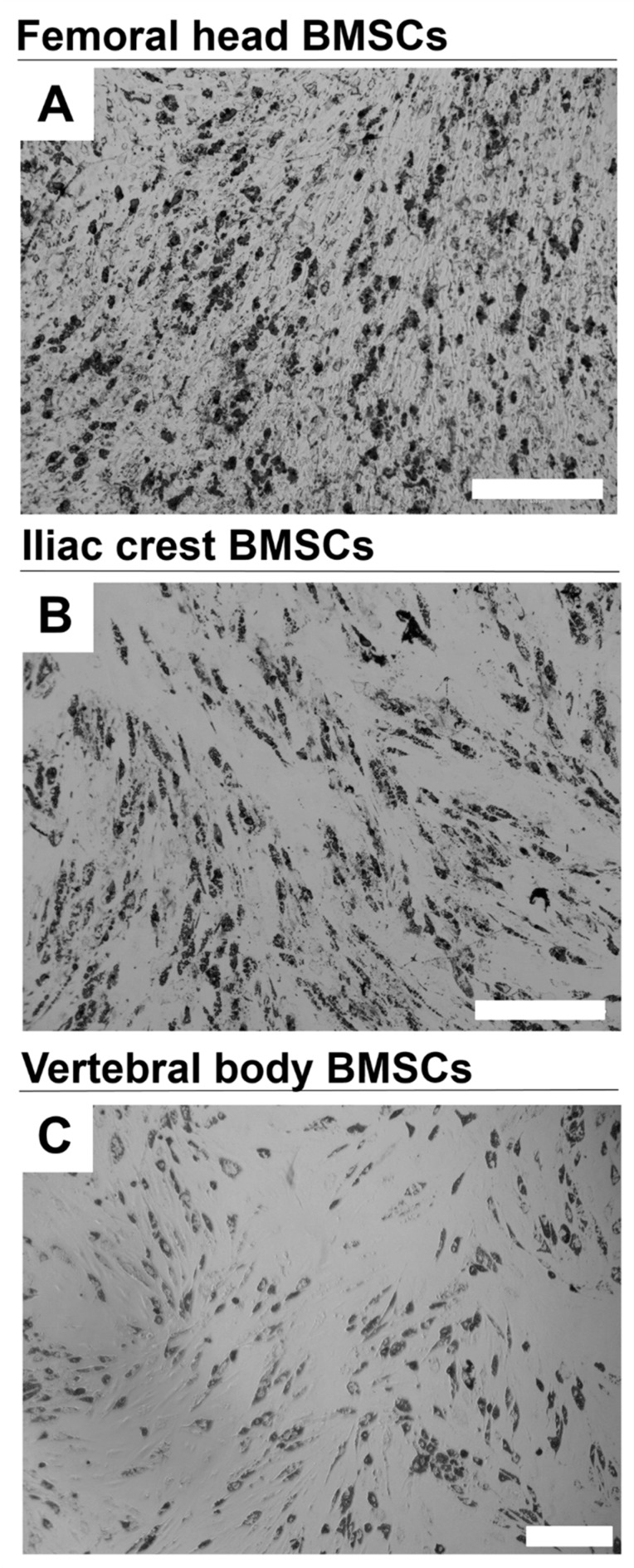
Adipogenic differentiation of BMSCs from different bone marrow sources. Representative images of adipogenically differentiated BMSCs from different bone marrow sources (**A**) femoral head, (**B**) iliac crest, (**C**) vertebral body was assessed by Oil Red O staining of deposited lipid droplets. Scale bars 400 µm. *n* = 10.

All BMSC sources readily differentiated toward the adipogenic lineage, as indicated by accumulation of lipid droplets staining positive for OilRed O (Figure 8).

## 3. Discussion

Inter-donor variability between BMSC cultures expanded in vitro poses major challenges not only in terms of understanding their basic biology but also for the development of BMSC-based therapies. This is inherent of endogenous progenitor cell heterogeneity and the lack of selection criteria that are functionally predictive. Moreover, progenitor populations remain insufficiently characterized with regard to anatomical site, gender, and age. Here, we investigated whether abundance of progenitor subsets found within the MNC fraction from bone marrow are phenotypically predictive for in vitro derived stromal cell cultures, i.e., if the higher abundance of one specific subpopulation correlates with better performance of the cell population derived from the MNC fraction. Therefore, we investigated progenitor abundance in the fresh MNC fraction and performed donor matched evaluations of growth kinetics and multi-lineage differentiation potential in vitro. In addition, we examined the influence of tissue source, gender, and age on derived stromal cell populations.

We hypothesized that the abundance of discrete progenitor populations in the freshly isolated MNC fraction may correlate with phenotypical variances in the derived stromal cells. For example, in the event that a particular progenitor population is more responsive to TGFβ driven chondrogenesis in vitro, should more of these cells be present in the original aspirate, it would be reasonable to presume that derived plastic adherent BMSCs may differentiate superiorly compared to a culture where fewer of these cells were originally present. To address this, we applied previously reported cell surface marker panels to freshly isolated MNCs targeting putative progenitor subsets: CD146+NG2+, CD45−CD34−CD146+, and CD45−CD34−CD73+.

Our data firstly revealed a large CD146+NG2+ population in comparison to less abundant CD45−CD34−CD73+ and CD45−CD34−CD146+ subsets. Rather than denoting a perivascular progenitor subset, it is likely that CD146+NG2+ cells comprise all pericytes, including the less abundant CD45−CD34−CD146+ progenitor sub-population. CD146 and NG2 are reported as in situ markers of the CD45−CD34−CD146+ perivascular cell population resident in adult tissues displaying plasticity in vitro and following in vivo transplantation [20]. Although frequently cited as pericyte markers, CD146 and NG2 are not specific to this cell type. CD146 (melanoma cell adhesion molecule) may be expressed by several cell types in bone marrow, such as endothelial cells, pericytes, and vascular smooth muscle cells [40]. NG2 (chondroitin sulphate proteoglycan 4) may be expressed by pericytes [41,42] and on macrophages [43]. Expression of both CD146 and NG2 on pericytes is dynamic and may be affected by the stage of angiogenesis, inflammation, and other external cues such as hypoxia [17,44,45]. Whilst endothelial cells may express CD146, they do not express NG2; therefore the likelihood of endothelial cell detection in our panel was small. Nevertheless, we cannot rule out the possibility that cell types other than pericytes were detected in the CD146+NG2+ panel. Secondly, we observed a positive correlation between the frequency of CD45−CD34−CD146+ and CD45−CD34−CD73+ mononuclear cells, suggesting that both marker panels may identify the same subpopulation of cells. Non-hematopoietic CD73+ cells are prevalent in bone marrow, and immunohistochemical studies indicate they occupy stromal and endosteal but not perivascular locations [16]. CD146+ is additionally expressed by reticular cells in the stromal compartment [16]. These studies together with our findings support the existence of a dual CD146+CD73+ progenitor subset likely resident in the stromal niche compartment of bone marrow.

As a field, we have yet to ascertain the developmental and the physiological basis for BMSC heterogeneity in bone marrow. Many groups have characterized progenitor populations both vascular and non-vascular associated in murine models and in man. If and how these populations are related in terms of ancestral heritage and function remains unclear. In 2008, Crisan and colleagues reported the in vitro multipotency of CD45-CD34-CD146+ cells sorted from multiple tissues including bone marrow [20]. The authors described this population to be equivalent to that of perivascular CD146+NG2+ cells or “pericytes”. Subsequent studies fueled the hypothesis that CD45−CD34−CD146+ cells may be bona fide progenitor cells in vivo and are the ancestor cells of all BMSCs [46]. Pericytes have historically been described as mural support cells surrounding the endothelium functioning to regulate capillary morphogenesis, stabilization, permeability, remodeling, and leukocyte trafficking [47,48]. Since it has not yet been demonstrated that all pericytes are progenitor cells, it appears that the CD45−CD34−CD146+ surface marker panel is not sufficient to draw any conclusion on the origin and the function of the derived cell populations. Also, the hypothesis of a universal multipotent MSC/pericyte cell population in virtually all vascularized tissues is challenged, since the multipotency of these cells could not always be confirmed in vivo [21,49]. Moreover, recent developmental lineage tracing and fate mapping studies in the mouse suggest that pericytes are heterogeneous in origin [50] and do not give rise to tissue specific progeny in the organs in which they reside [51].

These and other studies challenge the theory that BMSCs share a common ancestral source and rather suggest the existence of genetically and phenotypically distinct progenitor populations in adult bone marrow, a notion that was recently proposed as a concept of a “paralogous stem-cell niche” model [52].

A greater understanding of the biological nature and the function of these discrete progenitors is of paramount importance as we move forward to develop regenerative strategies for bone and articular cartilage, utilizing these progenitor cell populations or, in most cases, a mixture thereof. A deep knowledge of the regenerative potential of distinct cell population accompanied with straightforward isolation strategies will facilitate the success of these therapies.

A principle finding of our study was that, in terms of abundance, none of the putative progenitor cell subsets we detected by flow cytometry were predictive for the colony forming capacity or the proliferation ability of derived BMSC populations. Of the heterogeneous BMSC populations present in bone marrow, we know very little about which sub-populations are selected during plastic adhesion and monolayer expansion. Determining this is complicated by the fact that, once removed from their endogenous niche environment, BMSCs may alter cell-surface marker expression [9,10,11,12]. It may be that phenotypic drift during monolayer expansion overshadows any potential functional differences between progenitor subsets that may otherwise be predictive. Thus, it appears that, whilst cell surface markers can be useful in characterizing stromal progenitors endogenously, they are less useful for predicting the performance and indeed characterizing the in vitro derived culture. Improved characterization of non-hematopoietic progenitor populations in bone marrow may identify novel, more robust markers that can be used to select BMSCs on the basis of function. An alternative approach is to avoid monolayer expansion altogether. In vitro expansion on tissue culture plastic induces changes in BMSC morphology, gene and protein expression (including cell surface markers), growth kinetics, and plasticity [26,53,54,55,56,57,58,59]. Thus, in vitro derived BMSC cultures are neither genetically nor phenotypically representative of their in vivo counterparts [36]. The extent of phenotypic drift together with the clinical and the economic disadvantages to in vitro culture has prompted research aiming towards (i) intra-operative therapies using freshly isolated marrow cells and (ii) targeted approaches to initiate MSC homing from the bone marrow to sites of injury. There is therefore a need to better characterize freshly isolated bone marrow cell populations and discover predictive markers of their therapeutic efficacy in vivo. To this end, three-dimensional (3D) co-culture systems surpassing tissue culture plastic have been reported to maintain heterogeneity and cellular quiescence, allowing the study of bone marrow cells in a micro-environment more akin to that in vivo [38,60,61,62].

In the donors analyzed in this study, we did not observe significant effects of patient age or gender in terms of yield, growth kinetics, or multipotency of derived BMSC populations. At this point, we cannot exclude that this is attributed to a bias induced to the relatively small number of samples included in this study, presenting a major limitation of this work. The two best performing chondrogenic donors in our study were isolated from the iliac crest marrow of a female patient aged 23 and the vertebral body marrow of a male patient aged 61. Contrary findings may be found in the literature with some groups reporting differences in lifespan and/or growth kinetics between young and older patients [24,25,26,27,28,29,30]. A sex dimorphism has been reported in murine MSCs [63] and in man [30], although this area has yet to be fully investigated at the molecular level. As in many comparative BMSC studies, such conflicting findings may be attributed to sample size limitations together with differences in isolation/culture methods and outcome measurements. As in the majority of the aforementioned studies, our data were limited in that gender and age groups were disproportionately represented (10 female, 23 male; 10 < 50 yrs, 23 > 50 yrs).

Previous studies comparing different bone marrow sources have indicated higher CFU forming efficiency of cells isolated from vertebral body vs. iliac crest [33] but comparable multipotent differentiation potential [34]. Our data revealed no significant difference between anatomical sites in terms of BMSC yield normalized to total MNC fraction or the growth kinetics of derived stromal cell populations. In terms of absolute numbers, however, MNC number and MSC yields at p0 were consistently lower from the femoral head compared to the iliac crest and the vertebral body, which is in accordance with other studies [35]. We observed that donor variation between MSC derived from femoral head bone marrow was more prominent in terms of the relative abundance of progenitor cell subsets detected in the freshly isolated MNC fraction. This was reflected following culture expansion, where the percentage of CD146+ cells was also more variable when compared to vertebral body and iliac crest. Furthermore, among the samples analyzed in this study, MSC populations derived from femoral head marrow were overall inferior with respect to in vitro chondrogenesis compared to those derived from the iliac crest and the vertebral body. Specifically, pellet size and the level of sGAG and collagen protein staining appeared lower in responding chondrogenic donors from the femoral head. In the number of analyzed donors, our flow cytometry data did not show differences in terms of progenitor sub-population abundance when comparing femoral head to iliac crest and vertebral body. Thus, it appears that, independent of the abundance of these progenitor populations in the original tissue sample, femoral head derived stromal populations are more unpredictable and overall inferior when chondrogenically differentiated in vitro compared to vertebral body and iliac crest. Whilst this will have to be confirmed in future studies involving larger donor numbers, there could be several explanations for this. A greater proportion of femoral head derived progenitors may be more naive and/or not as responsive to TGFβ driven chondrogenesis. This could be investigated further by assessing stemness genes and the expression/activity of TGFβ family members. Alternatively, there may be a greater proportion of femoral head-derived progenitors that are lineage restricted. In addition, the variability in femoral head-derived cell populations might be attributable in part from variations in the microenvironment in which they reside, e.g., degenerated femoral head of patients undergoing hip replacement surgery due to multiple reasons ranging from fractures or femoral necrosis to primary or secondary coxarthrosis. In contrast, it can be hypothesized that the local microenvironment of BMSCs from other tissue sources investigated in this study might be less affected by the disease state of the individual donor.

In terms of clinical relevance, our findings suggest that the progenitor pool in the femoral head may not effectively support chondrogenesis in vivo. Indeed, femoral head marrow stimulation techniques aiming to recruit progenitors to the defect site result in variable, often unsatisfactory repair tissue that fails long term and is associated with poorer clinical outcomes compared to autologous chondrocyte implantation [64,65,66,67].

## 4. Materials and Methods

### 4.1. Bone Marrow Processing and Cell Isolation

Vertebral body, iliac crest, and femoral head bone marrow aspirates from patients undergoing spinal surgery/arthroplasty were acquired with informed consent and full ethical approval (KEK Bern 2016-00414 (12 April 2016), KEK-ZH-NR:2014-0420 (10 November 2014), EK Regensburg 12-101-0127 (12 June 2012). Bone marrow aspirates were processed from a total of 33 patients. This included 10 female and 23 male donors with an age range of 14–91; femoral head donors were aged 74 ± 13 years, iliac crest donors 43 ± 21 years, and vertebral body donors 63 ± 16 years (Table 1). Mononuclear cells (MNCs) were isolated from bone marrow by density centrifugation using Histopaque-1077™ (Sigma-Aldrich, Buchs, Switzerland) as described before [22,38,47]. Specifically, bone marrow diluted 1:4 in phosphate buffered saline (PBS) was layered onto Histopaque^®^-1077 centrifuged at 800× *g* for 20 min without braking. The resultant buffy coat containing the MNC fraction was aspirated using a Pasteur pipette and washed by two sequential centrifugations at 400× *g* for 15 min in cell culture media—alpha-MEM, 0.05% penicillin, and streptomycin (both GIBCO, Thermo Fisher Scientific, Zug, Switzerland) 10% foetal calf serum (FCS, Seraplus, Pan-Biotech, Aidenbach, Germany, P30-3700). A gated cell count was performed using a cell Scepter™ 2.0 Automated Cell Counter (Merck Millipore, Schaffhausen, Switzerland) to determine cell number > 8 µm (to gate out red blood cells and cell debris). MNCs were seeded at a density of 50,000 cells/cm^2^ into tissue culture flasks in cell culture media with the addition of fibroblast growth factor-2 at 5 ng/mL (Fitzgerald, Co. Wicklow, Ireland). FGF2 is a potent mitogen of BMSCs and is routinely used for monolayer expansion to maintain multipotency. Four days later, non-adherent hematopoietic cells were removed. Cells were passaged when colonies reached 80% confluency. The obtained cell count upon first passaging was recorded as an approximate measure of MSC frequency in MNCs. MSCs were expanded in monolayer (3 × 10^3^ cells/cm^2^) at 37 °C and 5% CO_2_ and received media changes three times a week. At each passage, population doublings were calculated using the following formula: LOG10 (final cell number/initial cell number)) × 3.33. MSCs were cultured until passage 2 (p2) and then transferred to liquid nitrogen for long term storage in cryopreservation media (FCS containing 8% DMSO (Sigma-Aldrich)).

### 4.2. Flow Cytometry Analysis

Flow cytometry analysis for hematopoietic and non-hematopoietic cell surface markers was performed on freshly isolated mononuclear cells and on adherent cell populations at p0 and p1. Cells were incubated with single or combinations of the following mouse monoclonal anti-human antibodies in PBS containing 1% fetal calf serum for 30 min at 4 °C: CD45-Alexa 488 (1:20, Beckman Coulter, Nyon, Switzerland), CD45-APC (1:5), CD73-PE-Cy7 (1:40), CD105-PE (1:40), CD90-BV421 (1:13), CD146-PeCy7 (1:20), HLA-DR-PE (1:5), CD19-PE (1:5), CD11b-PE (1:5, all BD Bioscience), CD44-APC (1:10), CD34-PE (1:10, both Miltenyi Biotec, Bergisch-Gladbach, Germany), NG2-PE (1:10), PDGF-rβ-Alexa 700 (1:20, both R&D Systems). Unstained isotype (IgG1-PeCy7 (1:20, BD Bioscience)) and single antibody controls were included. Flow cytometry was performed using a BD Aria III, and a minimum of 25,000 events were acquired for each sample, and data were analyzed using BD FACS Diva 6.1.3. Appropriate compensation settings and gating were applied in order to account for cellular debris, cell doublets, spectral overlap, and autofluorescence.

### 4.3. In Vitro Differentiation Assays

In vitro differentiation assays were performed using p2 BMSCs that were previously cryopreserved in liquid nitrogen.

#### 4.3.1. Chondrogenic Differentiation

For chondrogenic differentiation, 2.5 × 10^5^ MSCs were centrifuged at 500× *g* in 1.5 mL eppendorf tubes in chondrogenic induction media (DMEM high glucose medium, 0.11g/L sodium pyruvate (Sigma), 50 µg/mL ascorbic acid-2 phosphate (Sigma), 1% Insulin-Transferrin-Selenium + Premix (Corning, New York, NY, USA), 100 nM Dexamethasone (Sigma) and 10 ng/mL TGF-β-1 (Fitzgerald)). Culture media was replaced after 3 days and three times a week thereafter for a total of 21 days. Chondrogenic pellets were fixed in 70% methanol and snap-frozen, and cryosections were taken at 10 µm. Cryosections were stained with 0.1% Safranin-O and 0.02% Fast Green for detection of sulphated glycosaminoglycans (sGAG) or used for immunohistochemistry. Chondrogenic differentiation was assessed using a semi-quantitative score according to pellet size and the degree of Safranin-O staining (Table 3).

All steps during immunohistochemistry were performed at room temperature (RT) unless otherwise stated. Cryosections were blocked for 30 min in 0.3% hydrogen peroxide in methanol before treatment with 1 U/mL hyaluronidase (Sigma-Aldrich, cat: H3506) in phosphate buffered saline + 1% Tween (PBS-T) for 1 h at 37 °C. Sections were blocked with horse serum (Vector Laboratories, Burlingame, CA, USA, cat: S-2000) and diluted 1:20 in PBS-T for 1 h before incubation with (i) 5 μg/mL mouse monoclonal anti-collagen X (Affymetrix eBioscience, Thermo Fisher Scientific, cat: 14-9771) or (ii) mouse monoclonal anti-collagen II (DSHB, CIICI) diluted 1:20 in PBS-T. Secondary detection was performed using the Vectastain Elite ABC kit according to the manufacturer’s instructions (Vector Laboratories cat: PK-6100). Briefly, cryosections were washed, and biotinylated anti-mouse IgG secondary antibody was applied at 1:200 in PBS-T for 30 min (Vector Laboratories cat: BA-2001). After washing, sections were incubated with ABC complex for 30 min. Following another series of wash steps, ImmPACT DAB was applied for 4 min in the dark (Vector Laboratories, cat: SK-4105) prior to counterstaining with Mayer’s Haematoxylin (Fluka). Sections were dehydrated through a series of graded alcohols, cleared with xylene, and mounted with Eukitt mounting medium (Sigma). Sections were visualized and imaged using a Zeiss Axioplan 2 microscope (Oberkochen, Germany). Of note, the specificities of type II and type X antibodies were validated by testing on human articular cartilage and human scoliotic endplate, respectively (not shown).

#### 4.3.2. Osteogenic Differentiation

For osteogenic differentiation, cells were seeded in triplicates at a density of 20,000 cells/cm^2^ on thermanox coverslips (Thermo Fisher Scientific) in 24-well tissue culture plates and were either incubated in control medium (DMEM 1 g/L glucose, 10% FBS (Seraplus, Pan)) or osteogenic differentiation medium (DMEM 1 g/L glucose, 10% FBS, 50 µg/mL ascorbic acid, 5 mM glycerol-2-phosphate, 10 nM dexamethasone (all substances purchased from Sigma-Aldrich)). Cells were cultured for 21 days with three media changes per week. Mineral deposition was examined by alizarin red staining. For the staining, wells were washed twice with PBS, and cells were fixed for 15 min with 4% formaldehyde. After three washes with demineralized water, cells were stained with a 40 mM alizarin red S solution (Sigma-Aldrich) for 60 min. Excess stain was removed by repeated washing with demineralized water, and staining was visualized using an Axiovert40 CFL microscope (Zeiss). To compare osteogenic differentiation efficiency in-between different donors, stained wells were scanned using an EVOS^®^ FL Auto Cell Imaging System (Thermo Fisher Scientific), and the percentage of stained area was calculated in ImageJ (Rasband, NIH, Bethesda, MD, USA) by manually selecting cell-covered areas, transforming the image to grey values, and using an individual threshold to select for positively stained regions.

#### 4.3.3. Adipogenic Induction

For adipogenic differentiation, cells were seeded in triplicates at a density of 16,000 cells/cm^2^ in 24-well plates and incubated either in control medium (DMEM 4.5 g/L glucose, 10% FBS) or adipogenic differentiation medium (DMEM 4.5 g/L glucose, 10% FBS (Seraplus, Pan), 5 µg/mL insulin, 1 µM dexamethasone, 0.5 mM isobutylmethylxanthine, 60 µM indomethacine). Media were changed 3 times per week. After 2 weeks, wells were washed, fixed, and stained with oil red O solution (Sigma-Aldrich) to visualize lipid droplets. Stained plates were observed using an Axiovert40 CFL microscope.

### 4.4. Statistics

Data are presented from individual donors. Error bars indicate the standard deviation. Flow cytometry data are presented as means +/− standard deviations. Kruskal–Wallis analysis with Dunn’s multiple comparison test and Spearman rank correlation analysis was performed where appropriate using Prism software (GraphPad Software, La Jolla, CA, USA).

## 5. Conclusions

This study illustrates once more the large heterogeneity of bone marrow derived BMSC populations, which needs to be considered when making conclusions on the regenerative potential of these cells. Moreover, based on the donors included in the investigation, it appears that BMSCs derived from the femoral head were less abundant and performed inferiorly in terms of growth kinetics and chondrogenic differentiation compared to equivalent populations from the vertebral body and the iliac crest bone marrow. Since only a limited number of donors were assessed in these experiments, this needs to be confirmed in future studies. Contrary to our hypothesis, the presence of discrete progenitor cell subsets within the mononuclear cell fraction as identified by cell-surface marking profiling was not phenotypically predictive for derived BMSC cultures expanded in the monolayer. Demographically, neither gender nor age appeared to significantly influence the abundance or the multi-lineage differentiation potential of derived BMSCs. Considering our findings and those of others, although more clonogenic progenitor populations may be more readily derived from the marrow of younger patients, it is clear that the bone marrow of older individuals does indeed retain endogenous progenitors capable of multipotent differentiation in vitro and therefore represents an important adult stem cell pool for regenerative therapies. Greater characterization of resident tissue progenitors, for example, whole genome sequencing, micro-RNA, and epigenetic analysis, may help us better understand BMSC heterogeneity and the endogenous roles of these stromal progenitors in adult bone marrow. Furthermore, the development of 3D culture systems that better recapitulate the bone marrow microenvironment may negate the phenotypic drift experienced by BMSCs on tissue culture plastics, thus allowing us to better study naive BMSC biology. Ultimately, this knowledge will allow us to fine-tune BMSC behavior and develop more successful regenerative therapies for clinical practice.

## Figures and Tables

**Table 1:** Demographical information from patients where bone marrow samples were acquired. The total yield of mononuclear cells (MNCs) isolated by density centrifugation is shown. Bone marrow-derived stromal cells (BMSC) yield at p0/MNC originally seeded and the population doubling time (p0–p1) of tissue-culture plastic selected and monolayer expanded stromal cell populations.

Donor	Age	Sex	Tissue Source	MNCs Seeded	MSC Yield at p0/MNCs Seeded	MSC PDT (p0–p1)
1	86	Female	Femoral head	1.97 × 10^6^	0.30	0.69
2	67	Female	Femoral head	1.91 × 10^7^	0.19	0.77
3	86	Female	Femoral head	2.33 × 10^7^	0.39	0.77
4	51	Male	Femoral head	6.25 × 10^6^	0.58	1.00
5	91	Male	Femoral head	4.00 × 10^5^	0.51	0.91
6	67	Male	Femoral head	3.44 × 10^6^	0.06	0.73
7	46	Male	Femoral head	5.05 × 10^6^	0.95	0.94
8	83	Male	Femoral head	6.70 × 10^6^	0.13	0.35
9	81	Male	Femoral head	2.53 × 10^6^	0.38	0.65
10	83	Male	Femoral head	1.53 × 10^7^	0.12	0.70
11	75	Female	Femoral head	3.75 × 10^6^	0.95	0.55
12	61	Male	Femoral head	1.13 × 10^7^	0.46	0.57
13	68	Male	Femoral head	4.21 × 10^6^	0.24	0.85
14	83	Male	Femoral head	6.33 × 10^6^	0.14	0.80
15	82	Male	Femoral head	5.10 x 10^7^	0.14	0.24
16	88	Female	Iliac crest	4.35 × 10^7^	0.12	0.59
17	48	Female	Iliac crest	4.80 × 10^7^	0.25	0.86
18	46	Male	Iliac crest	3.20 × 10^7^	0.17	0.55
19	14	Male	Iliac crest	5.25 × 10^7^	0.16	0.61
20	41	Male	Iliac crest	7.84 ×10^6^	0.34	0.44
21	32	Male	Iliac crest	8.04 × 10^7^	0.39	0.73
22	23	Female	Iliac crest	1.43 × 10^7^	0.22	0.95
23	53	Male	Iliac crest	6.40 × 10^7^	0.09	0.57
24	75	Female	Vertebral body	6.40 × 10^7^	0.10	1.00
25	64	Female	Vertebral body	2.77 × 10^7^	0.73	0.65
26	39	Male	Vertebral body	1.60 × 10^7^	0.26	0.71
27	67	Male	Vertebral body	1.60 × 10^7^	0.12	0.44
28	46	Male	Vertebral body	3.20 × 10^7^	0.33	0.85
29	89	Male	Vertebral body	3.20 × 10^7^	0.14	0.70
30	77	Male	Vertebral body	1.60 × 10^7^	0.75	0.16
31	40	Male	Vertebral body	8.00 ×10^6^	0.32	0.78
32	60	Male	Vertebral body	3.20 × 10^7^	0.66	0.21
33	75	Female	Vertebral body	3.20 × 10^7^	0.13	0.52

**Table 2:** Expression of pericyte markers CD146 and NG-2 and MSC markers CD44, CD73, CD90, and CD105 in p0 and p1 BMSCs from different bone marrow sources was assessed by antibody staining and flow cytometry (given as % of positively stained cells).

		CD146	NG2	CD146/NG2	CD44	CD90	CD105	CD73	CD44/CD90 CD105/CD73
P0	fh	87.5 ± 11.9	86.0 ± 17.8	75.1 ± 19.1	100 ± 0.0	94.5 ± 3.6	100 ± 0.1	100 ± 0.0	94.5 ± 3.6
	ic	98.4 ± 1.3	88.7 ± 9.0	87.3 ± 9.1	100 ± 0.1	96.9 ± 1.1	99.90 ± 0.0	99.9 ± 0.1	96.9 ± 1.1
	vb	97.7 ± 1.0	65.7 ± 18.5	64.3 ± 17.8	100 ± 0.0	93.7 ± 4.8	99.98 ± 0.0	100 ± 0.0	93.7 ± 4.8
P1	fh	66.4 ± 22.7	74.7 ± 16.6	47.0 ± 9.5	100 ± 0.0	97.2 ± 1.5	99. 8 ± 0.3	100 ± 0.0	97.1 ± 1.7
	ic	97.4 ± 2.4	87.6 ± 13.0	85.2 ± 12.0	100 ± 0.1	96.6 ± 0.5	99.6 ± 0.5	99.7 ± 0.5	96.6 ± 0.4
	vb	95.1 ± 2.9	73.8 ± 13.6	72.7 ± 13.4	100 ± 0.1	93.7 ± 4.8	99.9 ± 0.3	100 ± 0.0	97.8 ± 2.2

Data are means ± standard deviation. fh: femoral head, ic: iliac crest, vb: vertebral body.

**Table 3:** Chondrogenic differentiation of bone marrow stromal cells assessed using a semi-quantitative histological score.

Chondrogenic Differentiation of Bone Marrow Stromal Cells					
Source	Age	Sex	Size	Safranin-O Staining	Score
Iliac crest	23	Female	+++	+++	6
Iliac crest	53	Male	+++	+	4
Iliac crest	45	Female	++	+	3
Femoral head	83	Male	++	+	3
Femoral head	75	Female	++	+	3
Femoral head	51	Male	+	++	3
Vertebral body	77	Male	++	+	3
Vertebral body	60	Male	++	+++	5
Vertebral body	48	Male	+++	+++	6

## Abbreviations

ARS	alizarin red S
BMSC	bone marrow stromal cell
CD	cluster of differentiation
CFU	colony forming unit
DMSO	dimethyl sulfoxide
FCS	foetal calf serum
FH	femoral head
IC	iliac crest
LepR	leptin receptor
MEM	minimum essential medium
MNC	mononuclear cell
MSC	mesenchymal stem cell or mesenchymal stromal cell
PBS	phosphate buffered saline
PDGF	platelet-derived growth factor
PDT	population doubling time
RT	room temperature
sGAG	sulphated glycosaminoglycans
TGF	transforming growth factor
VB	vertebral body
